# Supplementary material for: Measuring the tolerance of the genetic code to altered codon size
Source: eLife. 2022 Mar 16;11:e76941. doi: 10.7554/eLife.76941 (PMC9094753; doi:10.7554/eLife.76941)
Supplement: Supplementary file 1. — Canonical triplet tRNAs were cloned from Escherichia coli K12 genomic DNA. Body text Figures 2 and 3 concern mutations to the anticodon (bolded caps); body text Figure 4 concerns mutation to positions 32, 37, and 38 (bolded lower case). We use qtRNAthree letter scaffoldfour letter DNA codon nomenclature to refer to qtRNAs; for example, to create qtRNASerTAGA, the CGA anticodon in tRNASer scaffold would be replaced by TCTA. [file elife-76941-supp1.docx]

**Supplementary File 1 - Table of tRNA scaffolds**

| **tRNA** | **Sequence** (bold caps = anticodon;  bold = positions 32, 37, 38) | **Expression plasmid map**  (IPTG inducible in S2060 strain) |
| --- | --- | --- |
| tRNA^Ala^ | ggggctatagctcagctgggagagcgcttgc**a**t**GGCat**gcaagaggtcagcggttcgatcccgcttagctccacca | <https://benchling.com/s/seq-tg2Ok8ejpnqNNR1RCgxJ> |
| tRNA^Arg^ | gcatccgtagttcagctggatagagtactcgg**c**t**ACGaa**ccgagcggtcggaggttcgaatcctcccggatgcacca | <https://benchling.com/s/seq-5Z1JRvAM5CUKFL2CyQHl> |
| tRNA^Asn^ | tcctctgtagttcagtcggtagaacggcgga**c**t**GTTaa**tccgtatgtcactggttcgagtccagtcagaggagcca | <https://benchling.com/s/seq-cvpe4WFNdq3urdBOErUE> |
| tRNA^Asp^ | ggagcggtagttcagtcggttagaatacctgc**c**t**GTCac**gcagggggtcgcgggttcgagtcccgtccgttccgcca | <https://benchling.com/s/seq-cvcp7xMH4VVbQxCWY57a> |
| tRNA^Cys^ | ggcgcgttaacaaagcggttatgtagcgga**t**t**GCAaa**tccgtctagtccggttcgactccggaacgcgcctcca | <https://benchling.com/s/seq-pCOWJ5Ba9vyLDMfhXXTr> |
| tRNA^Glu^ | gtccccttcgtctagaggcccaggacaccgcc**c**t**TTCac**ggcggtaacaggggttcgaatcccctaggggacgcca | <https://benchling.com/s/seq-cYNon2B0YXiyMus5Egj2> |
| tRNA^Gln^ | tggggtatcgccaagcggtaaggcaccgga**t**t**CTGat**tccggcattccgaggttcgaatcctcgtaccccagcca | <https://benchling.com/s/seq-1iWjqPpZE7TQZ4cWEbKI> |
| tRNA^Gly^ | gcgggcgtagttcaatggtagaacgagagc**t**t**CCCaa**gctctatacgagggttcgattcccttcgcccgctcca | <https://benchling.com/s/seq-XFAUplPfQA8J3y3eKe9o> |
| tRNA^His^ | gtggctatagctcagttggtagagccctgga**t**t**GTGat**tccagttgtcgtgggttcgaatcccattagccacccca | <https://benchling.com/s/seq-xS0QHSq6wIqNBGG12Vxl> |
| tRNA^Ile^ | aggcttgtagctcaggtggttagagcgcaccc**c**t**GATaa**gggtgaggtcggtggttcaagtccactcaggcctacca | <https://benchling.com/s/seq-cUTbLsat2L7hQcjOZxXW> |
| tRNA^Leu^ | gccgaagtggcgaaatcggtagacgcagttga**t**t**CAAaa**tcaaccgtagaaatacgtgccggttcgagtccggccttcggcacca | <https://benchling.com/s/seq-PSGYeWnss5uNAMjGNWk9> |
| tRNA^Lys^ | gggtcgttagctcagttggtagagcagttga**c**t**TTTaa**tcaattggtcgcaggttcgaatcctgcacgacccacca | <https://benchling.com/s/seq-vsDR689MSNPR3k4lsy8l> |
| tRNA^Met^ | ggctacgtagctcagttggttagagcacatca**c**t**CATaa**tgatggggtcacaggttcgaatcccgtcgtagccacca | <https://benchling.com/s/seq-NUddULsETeJ7xYfqySrf> |
| tRNA^fMet^ | cgcggggtggagcagcctggtagctcgtcggg**c**t**CATaa**cccgaaggtcgtcggttcaaatccggcccccgcaacca | <https://benchling.com/s/seq-kKD5TUaRnzoDXYhJBfdf> |
| tRNA^Phe^ | gcccggatagctcagtcggtagagcagggga**t**t**GAAaa**tccccgtgtccttggttcgattccgagtccgggcacca | <https://benchling.com/s/seq-W61IkhltEYkNdtNOTG5h> |
| tRNA^Pro^ | cggtgattggcgcagcctggtagcgcacttcg**t**t**CGGga**cgaaggggtcggaggttcgaatcctctatcaccgacca | <https://benchling.com/s/seq-2vdHeJ6LQJyDgaX3uiZv> |
| tRNA^Ser^ | ggagagatgccggagcggctgaacggaccggt**c**t**CGAaa**accggagtaggggcaactctaccgggggttcaaatccccctctctccgcca | <https://benchling.com/s/seq-cGi0J8BtBUydV1m1uq2S> |
| tRNA^Thr^ | gccgatatagctcagttggtagagcagcgca**t**t**CGTaa**tgcgaaggtcgtaggttcgactcctattatcggcacca | <https://benchling.com/s/seq-QR5vD3dJn5S23IbSXpMp> |
| tRNA^Trp^ | aggggcgtagttcaattggtagagcaccggt**c**t**CCAaa**accgggtgttgggagttcgagtctctccgcccctgcca | <https://benchling.com/s/seq-PsQbqBCffQ1CVLU6tdlK> |
| tRNA^Tyr^ | ggtggggttcccgagcggccaaagggagcaga**c**t**GTAaa**tctgccgtcacagacttcgaaggttcgaatccttcccccaccacca | <https://benchling.com/s/seq-8fpqOoBdg536ImikNeYy> |
| tRNA^Val^ | gcgtccgtagctcagttggttagagcaccacc**t**t**GACat**ggtgggggtcggtggttcgagtccactcggacgcacca | <https://benchling.com/s/seq-LROPe63eAegmXN9pKMWp> |

Canonical triplet tRNAs were cloned from *E. coli* K12 genomic DNA. Body text Figures 2 and 3 concern mutations to the anticodon (bolded caps); body text Figure 4 concerns mutation to positions 32, 37 and 38 (bolded lower case). We use qtRNA^three letter scaffold^_four letter DNA codon_ nomenclature to refer to qtRNAs; e.g. to create qtRNA^Ser^_TAGA_, the CGA anticodon in tRNA^Ser^ scaffold would be replaced by TCTA.
