# Supplementary material for: Measuring the tolerance of the genetic code to altered codon size
Source: eLife. 2022 Mar 16;11:e76941. doi: 10.7554/eLife.76941 (PMC9094753; doi:10.7554/eLife.76941)
Supplement: Supplementary file 2. — For sfGFP purification, qtRNA expression plasmids (as listed) were co-expressed with C-terminal 6xHis-tagged sfGFP with the appropriate quadruplet codon replacing permissive residue 151. For example, sfGFP-151-GGGG,https://benchling.com/s/seq-bl1bixktGKegGwboMYIP. Peptides detected are listed in Supplementary file 3. Raw spectra have been deposited in the PRIDE database (Perez-Riverol et al., 2022), dataset identifier PXD031925 and 10.6019/PXD031925. [file elife-76941-supp2.docx]

**Supplementary File 2 - purification scale of qtRNA-translated proteins**

| **qtRNA** | **Purification scale** | **Result** | **Amino Acid Incorporation** | **Limit of detection** | **Expression plasmid map**  (IPTG inducible in S2060 E. coli strain) |
| --- | --- | --- | --- | --- | --- |
| Ala-AGGG-32T-38G | 1L | Purified and measured sample | 77% Arg;  22% Ala | 0.002% | <https://benchling.com/s/seq-MDPm0wgpoZcbQbgcrecA> |
| Asn-AGGA | 1L | Yield too low to measure | - | - | <https://benchling.com/s/seq-xWG68x7wR1CshAE1tV9l> |
| Asp-CGGC-32G-38A | 1L | Purified and measured sample | 100% Arg | 0.002% | <https://benchling.com/s/seq-7thPCofT0Jhr9UB7h4OL> |
| Cys-CGGC | 1L | Yield too low to measure | - | - | <https://benchling.com/s/seq-rHgdjGw6XdyEYp84L6J7> |
| Gln-CAGG | 4mL | Purified and measured sample | 100% Gln | 0.002% | <https://benchling.com/s/seq-tGyQojadJ2mRCj41l54O> |
| Glu-CGGT | 4mL | Purified and measured sample | 100% Glu | 0.1% | <https://benchling.com/s/seq-nr3CInJvm9MWjnGcvy4s> |
| Gly-GGGG | 4mL | Purified and measured sample | 100% Gly | 0.0003% | <https://benchling.com/s/seq-Ry0fYxIuIoL0YvXj8XxQ> |
| His-TACA-32G | 1L | Purified at 1L scale, but no peptide detected in mass spec | - | - | <https://benchling.com/s/seq-HOf86rVgpat27IDVvCxY> |
| Ile-AGGA | 1L | Purified and measured sample | 100% Arg | 0.005% | <https://benchling.com/s/seq-hbgRy8yUWsOtbdbpmdP1> |
| Leu-AGGG | 1L | Yield too low to measure | - | - | <https://benchling.com/s/seq-V8jP2xPq1V9vI0Ps3cHl> |
| Lys-AGAA | 1L | Yield too low to measure | - | - | <https://benchling.com/s/seq-fyjM4W7dLZl6RBOHKzwr> |
| Met-AGGG | 1L | Purified and measured sample | 96% Arg;  3% Met;  2% Gly | 0.002% | <https://benchling.com/s/seq-T9ehIv5N1loIyU4sX9V7> |
| Phe-CGGC | 4mL | Purified and measured sample | 100% Phe | 0.8% | <https://benchling.com/s/seq-fgpEdowxfsOdeuYLgs5V> |
| Pro-CCGG | 1L | Purified and measured sample | 100% Pro | 0.009% | <https://benchling.com/s/seq-T1vdy3JSiZ2DNG91TwA6> |
| Ser-TCGG-32A-38C | 1L | Purified and measured sample | 100% Ser | 0.04% | <https://benchling.com/s/seq-TWCRTU1TcjS0u19SnKEW> |
| Thr-ACGG | 1L | Yield too low to measure | - | - | <https://benchling.com/s/seq-cJDL7NuzsBDMZnkLzEpL> |
| Trp-AGGG | 1L | Purified and measured sample | 100% Arg | 0.2% | <https://benchling.com/s/seq-g8megLDGGL2LlvGVyJFi> |
| Val-CGGC-32T-38A | 1L | Purified and measured sample | 100% Arg | 0.005% | <https://benchling.com/s/seq-i3tcy29Qtmv8ru3xLvb5> |
| Tyr-, Arg-, and other TAGA qtRNAs[^9^](https://paperpile.com/c/ETNcpJ/sOCa) | 4mL | Purified and measured sample | Assorted | Assorted |  |

For sfGFP purification, qtRNA expression plasmids (as listed) were co-expressed with C-terminal 6xHis-tagged *sfGFP* with the appropriate quadruplet codon replacing permissive residue 151. For example, sfGFP-151-GGGG, <https://benchling.com/s/seq-bI1bixktGKegGwboMYIP>. Peptides detected are listed in Supplementary File 3. Raw spectra have been deposited in the PRIDE database[^24^](https://paperpile.com/c/ETNcpJ/o2uC), dataset identifier PXD031925 and 10.6019/PXD031925.
