# Supplementary material for: Measuring the tolerance of the genetic code to altered codon size
Source: eLife. 2022 Mar 16;11:e76941. doi: 10.7554/eLife.76941 (PMC9094753; doi:10.7554/eLife.76941)
Supplement: Supplementary file 3. — List of peptides flanking residue 151 that were detected during mass spectrometry. Raw spectra have been deposited in the PRIDE database (Perez-Riverol et al., 2022), dataset identifier PXD031925 and 10.6019/PXD031925. [file elife-76941-supp3.docx]

**Supplementary File 3 - Mass spectrometry of peptides flanking residue 151**

List of peptides flanking residue 151 that were detected during mass spectrometry. Raw spectra have been deposited in the PRIDE database[^24^](https://paperpile.com/c/ETNcpJ/o2uC), dataset identifier PXD031925 and 10.6019/PXD031925.

| **qtRNA** | **Highest AUC** | **Limit of detection** | **Peptides detected** | **AUC of peptide** |
| --- | --- | --- | --- | --- |
| Ala-AGGG-32T-38G | 6.72E+08 | 0.0015% |  |  |
|  |  |  | K.LEYNFNSHNVA(sub Y)ITADKQK.N | 3.42E+07 |
|  |  |  | K.LEYNFNSHNVA(sub Y)ITADK.Q | 9.92E+07 |
|  |  |  | K.LEYNFNSHNVR(sub Y).I | 6.72E+08 |
|  |  |  | K.LEYNFD(sub N)SHNVR(sub Y).I | 1.16E+07 |
|  |  |  | L.EYNFNSHNVR(sub Y).I | 9.96E+07 |
|  |  |  | E.YNFNSHNVR(sub Y).I | 1.26E+08 |

| Asp-CGGC-32G-38A | 5.19E+08 | 0.0019% |  |  |
| --- | --- | --- | --- | --- |
|  |  |  | I.LGHKLEYNFNSHNVR(sub Y).I | 2.27E+07 |
|  |  |  | H.KLEYNFNSHNVR(sub Y).I | 8.10E+05 |
|  |  |  | K.LEYNFNSHNVR(sub Y).I | 5.19E+08 |

| Gln-CAGG | 6.68E+08 | 0.0015% |  |  |
| --- | --- | --- | --- | --- |
|  |  |  | K.LEYNFNSHNVQ(sub Y)ITADKQK.N | 6.68E+08 |

| Glu-CGGT | 3.78E+07 | 0.0265% |  |  |
| --- | --- | --- | --- | --- |
|  |  |  | K.LEYNFNSHNVE(sub Y)ITADK.Q | 1.25E+07 |
|  |  |  | K.LEYNFNSHNVE(sub Y)ITADKQK.N | 3.78E+07 |

| Gly-GGGG | 3.44E+08 | 0.0003% |  |  |
| --- | --- | --- | --- | --- |
|  |  |  | K.LEYNFNSHNVG(sub Y)ITADK.Q | 1.85E+08 |
|  |  |  | K.LEYNFNSHNVG(sub Y)ITADKQK.N | 3.44E+08 |

| Ile-AGGA | 1.29E+07 | 0.0775% |  |  |
| --- | --- | --- | --- | --- |
|  |  |  | K.LEYNFNSHNVR(sub Y).I | 1.29E+07 |

| Met-AGGG | 6.84E+08 | 0.0015% |  |  |
| --- | --- | --- | --- | --- |
|  |  |  | K.LEYNFNSHNVG(sub Y)ITADK.Q | 5.96E+06 |
|  |  |  | K.LEYNFNSHNVR(sub Y).I | 6.84E+08 |
|  |  |  | K.LEYNFNSHNVM(sub Y)ITADK.Q | 1.44E+07 |
|  |  |  | K.LEYNFNSHNVM(sub Y)ITADKQK.N | 4.93E+06 |
|  |  |  | K.LEYNFDSHNVR(sub Y).I | 6.56E+06 |

| Phe-CGGC | 1.25E+06 | 0.8000% |  |  |
| --- | --- | --- | --- | --- |
|  |  |  | K.LEYNFNSHNVF(sub Y)ITADK.Q | 1.25E+06 |

| Pro-CCGG | 1.06E+08 | 0.0094% |  |  |
| --- | --- | --- | --- | --- |
|  |  |  | K.LEYNFNSHNVP(sub Y)ITADK.Q | 1.06E+08 |
|  |  |  | K.LEYNFNSHNVP(sub Y)ITADKQK.N | 2.33E+07 |

| Ser-TCGG-32A-38C | 2.60E+07 | 0.0385% |  |  |
| --- | --- | --- | --- | --- |
|  |  |  | K.LEYNFNSHNVS(sub Y)ITADKQK.N | 2.60E+07 |

| Trp-AGGG | 4.88E+06 | 0.2049% |  |  |
| --- | --- | --- | --- | --- |
|  |  |  | K.LEYNFNSHNVR(sub Y).I | 4.88E+06 |

| Val-CGGC-32T-38A | 1.90E+08 | 0.0053% |  |  |
| --- | --- | --- | --- | --- |
|  |  |  | K.LEYNFNSHNVR(sub Y).I | 1.90E+08 |
